# Fucose Binding Cancels out Mechanical Differences between Distinct Human Noroviruses

**DOI:** 10.3390/v15071482

**Published:** 2023-06-30

**Authors:** Yuzhen Feng, Ronja Pogan, Lars Thiede, Jürgen Müller-Guhl, Charlotte Uetrecht, Wouter H. Roos

**Affiliations:** 1 Moleculaire Biofysica, Zernike Instituut, Rijksuniversiteit Groningen, 9747AG Groningen, The Netherlands; yuzhen.feng@rug.nl; 2 CSSB Centre for Structural Systems Biology, Deutsches Elektronen-Synchrotron (DESY) & Leibniz Institute of Virology (LIV), 22607 Hamburg, Germany; ronja.pogan@cssb-hamburg.de (R.P.); lars.thiede@cssb-hamburg.de (L.T.); juergen.mueller@leibniz-liv.de (J.M.-G.); charlotte.uetrecht@cssb-hamburg.de (C.U.); 3 Faculty V: School of Life Sciences, University of Siegen, 57076 Siegen, Germany; 4 Partner Site Hamburg-Lübeck-Borstel-Riems, Bernhard Nocht Institute for Tropical Medicine and German Center for Infection Research (DZIF), 20359 Hamburg, Germany

**Keywords:** AFM, nanoindentation, norovirus-like particles (noroVLPs), mechanical properties, virus-ligand interaction

## Abstract

The majority of nonbacterial gastroenteritis in humans and livestock is caused by noroviruses. Like most RNA viruses, frequent mutations result in various norovirus variants. The strain-dependent binding profiles of noroviruses to fucose are supposed to facilitate norovirus infection. It remains unclear, however, what the molecular mechanism behind strain-dependent functioning is. In this study, by applying atomic force microscopy (AFM) nanoindentation technology, we studied norovirus-like particles (noroVLPs) of three distinct human norovirus variants. We found differences in viral mechanical properties even between the norovirus variants from the same genogroup. The noroVLPs were then subjected to fucose treatment. Surprisingly, after fucose treatment, the previously found considerable differences in viral mechanical properties among these variants were diminished. We attribute a dynamic switch of the norovirus P domain upon fucose binding to the reduced differences in viral mechanical properties across the tested norovirus variants. These findings shed light on the mechanisms used by norovirus capsids to adapt to environmental changes and, possibly, increase cell infection. Hereby, a new step towards connecting viral mechanical properties to viral prevalence is taken.

## 1. Introduction

Norovirus, which belongs to the family *Caliciviridae*, is a non-enveloped, single-stranded RNA virus [1]. Human norovirus causes the majority of non-bacterial gastroenteritis outbreaks worldwide [2]. Human norovirus infection is self-limiting in healthy individuals, usually lasting for several days, but is associated with severe complications in immunocompromised individuals [3]. Although target cells for norovirus infection have not been well defined yet, active viral replication has been found in the enterocytes and enteroendocrine cells of the small intestine. Here, abundant human blood group antigens (HBGAs) are present on the cell surfaces [4]. Fucose moieties of HBGAs are regarded as the primary determinant for norovirus recognition [5], and several fucose binding pockets have been mapped on the norovirus capsid protein [6]. After passing through the intestinal tract with different pH values and ionic concentrations at each portion, norovirus reaches the small intestine, attaches to fucose moieties on the surfaces of its target cells, and putatively initiates infection through a yet unknown protein receptor [7,8].

Like other RNA viruses, frequent mutations generate a high diversity of noroviruses, with ten genogroups recognized today (GI–GX), including GI, GII, GIV, and GVIII, which are infectious to humans. Each genogroup can be further divided into various genotypes based on the capsid amino acid sequences [9]. Nowadays, newly emerging strains, especially from the GII, have replaced the rarely found prototypical GI.1 Norwalk. Recent GII.17 Kawasaki has been considered a predominant strain in East Asia temporarily. It remains to be seen whether it has the potential to overcome the currently predominant GII.4 norovirus [10]. GII.17 Kawasaki showed a distinct stability pattern from GI.1 Norwalk upon pH changes in native mass spectrometry [11]. Compared to GII.17 Kawasaki, GII.10 Vietnam is still a comparably rare strain at the moment. However, it shows broad interactions with all five secreted HBGAs, including A, B, H, Leb, and Ley antigens. This wide binding profile with viral attachment factors raises concern about the possible prevalence of GII.10 Vietnam [12].

**Figure 1:**
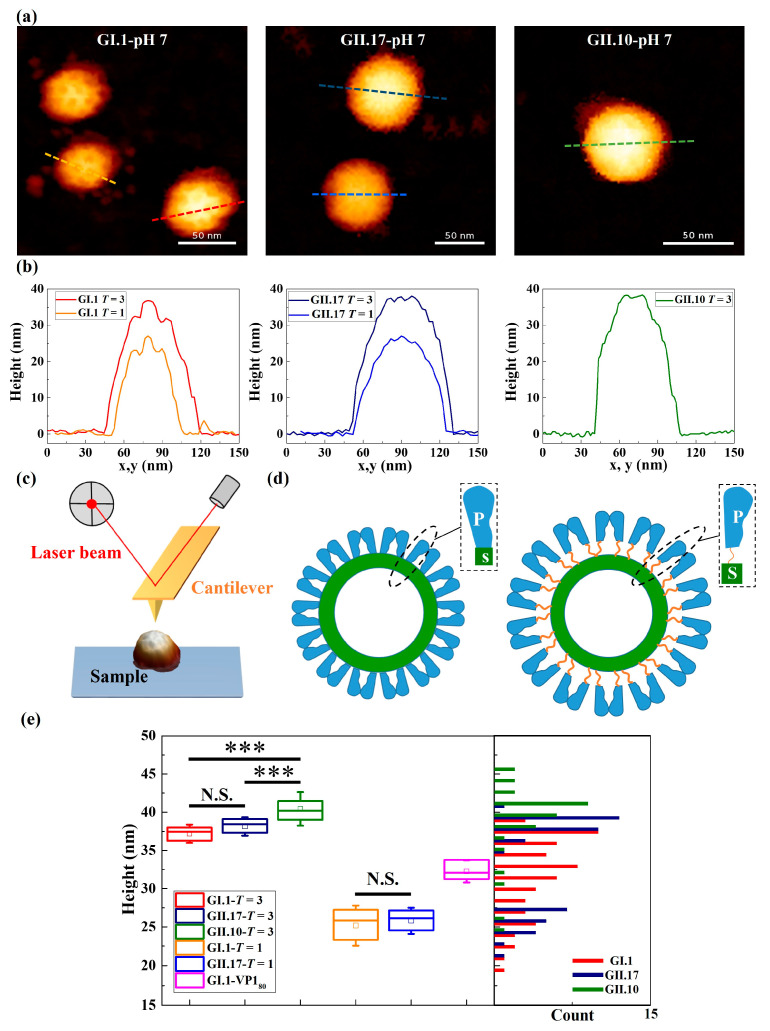
Representative images of tested noroVLPs of norovirus variants and the comparison of their sizes. (**a**) AFM images and (**b**) the corresponding height profiles of GI.1, GII.17, and GII.10 at pH 7.0. The height profiles were taken at the dashed lines of the respective colour in the corresponding images. The protruding outer layer and the inner layer were depicted by the contrast of light and dark on the surface of particles in AFM images and the protrusions and depressions on height profiles, respectively. The height scale of AFM images is 40 nm. (**c**) Scheme of the AFM setup for studying noroVLPs. The AFM cantilever with a sharp tip at its end was used for imaging and indenting an individual noroVLP immobilized on a hydrophobic glass cover slip. A laser beam is focused on the rear side of the cantilever to detect its deflection. (**d**) Scheme of noroVLPs whose P domain of VP1 is at resting (**left**) and rising (**right**) conformation. The flexible hinge region connecting a P domain and a S domain is depicted as an orange string. (**e**) The corrected height distribution of tested noroVLPs. The corrected heights of noroVLPs of each norovirus variant were acquired from three independent measurements. Assembly types of tested noroVLPs were assigned based on the size distribution of GII.17. The noroVLPs with corrected height less than 29 nm were assigned as *T* = 1 assembly. The noroVLPs that are higher than 34 nm were assigned as *T* = 3 assembly. The whiskers in the box plot represent the standard deviation. Significant differences between *T* = 3 assemblies of variants were assessed using a one-way ANOVA test with Bonferroni post hoc analysis, and significant differences between *T* = 1 assemblies of variants were assessed using the Brown–Forsythe test. P values are indicated by asterisks: *p* < 0.001 (***) and not significant (N.S.).

The human norovirus capsid is composed of the major capsid protein VP1 and the minor capsid protein VP2. 90 VP1 dimers assemble into the typical *T* = 3 viral capsid. VP2 is present in virions and carries multiple functions that are proposed to enhance VP1 expression levels. However, VP2 is generally not incorporated into virus-like particles (VLPs). By expressing VP1 in insect cells, noroVLPs can be assembled with comparable morphology and antigenic properties as virions [13]. VP1 can be divided into a shell (S) domain and a protruding (P) domain connected by a flexible hinge. As shown in Figure 1d, the S domain forms the inner spherical shell, and the P domain forms the outer protruding arches emanating from the inner spherical shell. These P domains have two distinct conformations, which can be observed sometimes even within the same genotype [14,15,16,17]. The difference between these conformations can be described by an extension or retraction of the hinge region, resulting in a resting or rising conformation. A dynamic switch between the two conformations has been observed for both murine [16] and human norovirus GII.4 [18].

Identifying the virus causing an infection is often the first and most important step in designing a targeted treatment. Several methods have been used for virus identification, assessing various parameters of the virus. For instance, cell culture-based approaches examine cytopathic effects [19], molecular approaches quantify viral nucleic acid and protein [20], and microscopy techniques characterize the size and morphology of the virus [21]. However, frequent mutations of viruses impose challenges on the current virus identification methods, and a novel complementary approach is demanded. In this study, using AFM nanoindentation technology [22,23,24] and native mass spectrometry (native MS) [25,26], we investigated noroVLPs of GI.1 Norwalk, GII.17 Kawasaki 308, and GII.10 Vietnam 026. The mechanical properties of noroVLPs of these variants were measured under neutral conditions, alkaline conditions, and after fucose treatment, respectively. By AFM experiments, we found the noroVLP deformation of tested variants followed a stepwise manner. Both the mechanical properties and assembly types of noroVLPs demonstrated differences under neutral conditions. Furthermore, significant differences were found in response to fucose treatment. The different variants ended up with similar mechanical properties due to their distinct responses to fucose binding. Our findings provide new insights into the viral mechanical properties and possibly divergent infection strategies of norovirus variants. Significant differences in viral mechanical properties and assembly types among these three strains addressed by AFM nanoindentation and native MS propose a possible approach to identifying viruses based on their physical properties.

## 2. Materials and Methods

### 2.1. NoroVLP Production and Purification

To produce noroVLPs, full-length VP1 genes were cloned and expressed in insect cells using a baculovirus system as described previously [27,28]. GenBank accession numbers are AY602016.1, AF504671.2, and LC037415.1 for variants GI.1 Norwalk, GII.10 Vietnam 026, and GII.17 Kawasaki 308, respectively. In brief, recombinant VP1 bacmid was transfected into Sf9 insect cells and incubated for 5–7 days at 27 °C. After incubation, the culture medium was collected and centrifuged for 10 min at 3000 rpm at 4 °C. Then, the supernatant containing the recovered baculovirus was used to infect Hi5 insect cells and incubated for 5 days at 27 °C. After this incubation, secreted noroVLPs were acquired from the culture medium by first centrifuging for 10 min at 3000 rpm at 4 °C and then for 1 h at 6500 rpm at 4 °C. Subsequently, the obtained supernatant was concentrated by ultracentrifugation (Ti45 rotor, Beckman, Brea, CA, USA) at 35,000 rpm for 2 h at 4 °C to become concentrated noroVLPs. Next, the concentrated noroVLPs were purified using CsCl equilibrium gradient ultracentrifugation at 35,000 rpm (SW56 rotor, Beckman) for 18 h at 4 °C. Finally, noroVLPs were pelleted for 2 h at 40,000 rpm (TLA55 rotor, Beckman) at 4 °C and dissolved in PBS buffer at pH 7.4.

### 2.2. AFM Sample Preparation

Neutral and alkaline 250 mM ammonium acetate (99.999% trace metal basis, Sigma-Aldrich, St. Louis, MO, USA) solutions were freshly prepared before AFM experiments. For the measurements under neutral and alkaline conditions, the pH value of ammonium acetate solution was adjusted by acetic acid (Sigma-Aldrich) and ammonium hydroxide (Sigma-Aldrich) to pH 7.0 and pH 9.0, respectively. Prior to AFM measurements, the stock solutions of noroVLPs were diluted to a concentration of VP1 monomers of 0.1 μM. To immobilize noroVLPs during AFM measurements, a hydrophobic glass cover slip was applied as a substrate. The hydrophobic coating on the glass cover slip was obtained by applying a hexamethyldisilazane (Sigma-Aldrich) treatment as described previously [29,30]. A droplet of 200 μL diluted noroVLP solution was deposited on a hydrophobic glass cover slip. After 15 min of incubation at room temperature, another 1 mL of neutral or alkaline solution was added to a liquid receptacle for follow-up AFM imaging and nanoindentation.

### 2.3. Fucose Treatment

Glycan binding to norovirus has been investigated using several biophysical approaches, and dissociation constants (K_d_) in the μM range and even as low as mM have been found [31,32]. The K_d_ of methyl α-L-fucopyranoside (referred to as fucose in the following) to GII.4 Saga P-dimers is 22 mM [33]. The HBGA binding sites of GII.10 and GII.4 are almost identical [34]. Therefore, this K_d_ was used as an estimate for the K_d_ of fucose in the tested norovirus variants. For reaching a high occupancy (90% occupancy) of fucose binding sites at a final concentration of VP1 monomers of 0.1 μM, we used this K_d_ to calculate the fucose concentration. This resulted in incubating noroVLPs with 198 mM methyl α-L-fucopyranoside (Carbosynth, Bratislava, Slovakia) in a 250 mM ammonium acetate solution (pH 7.0) for 15 min. After incubation, a droplet of 200 μL of the solution containing noroVLPs and bound fucose was deposited onto a hydrophobic glass cover slip to immobilize noroVLPs. After 15 min of incubation, another 1 mL of 250 mM ammonium acetate solution (pH 7.0) was added to a liquid receptacle, yielding a final concentration of fucose of 40 mM during AFM imaging and nanoindentation.

### 2.4. AFM Imaging and Nanoindentation

The experimental setup is schematically shown in Figure 1c. Imaging and nanoindentation experiments on noroVLPs were conducted by AFM (NanoWizard, JPK). All experiments were performed in liquid. Before AFM imaging, the cantilever (qp-Bio AC CB3, Nanosensors) with a nominal spring constant of 0.05 N/m and a typical tip radius of curvature smaller than 10 nm was calibrated using the contact-based method. The parameters for imaging noroVLPs in quantitative imaging (QI) mode were kept constant and set as follows: The setpoint was 0.12 nN; the Z length was 30 nm; the pixel time was 8–10 ms; and the resolution of the scan area was 128 × 128 pixels.

After imaging the noroVLPs by QI imaging, nanoindentation was performed at the center of a noroVLP. The nanoindentation procedure was carried out according to the previous protocol [30]. The settings for nanoindentation were: Z length 200 nm; setpoint 2 nN; loading rate 50 nm/s. Before and after indenting the targeted noroVLP, indentations were performed on the glass substrate adjacent to the noroVLP to confirm the cleanliness of the tip. Finally, the noroVLP was imaged again to visualize the structural state of the particle after nanoindentation.

### 2.5. Data Processing

In the Force-Displacement (F-Z) curve obtained from AFM nanoindentation, the slope of the linear part represents the combined force responses of an AFM cantilever and noroVLP, giving the total spring constant of the cantilever-particle system ($k_{total}$). The spring constant of the cantilever ($k_{cantilever}$) was acquired by the calibration protocol. Using Hooke’s law for two springs in a series, the spring constant of noroVLP ($k_{virus}$) is calculated by the following equation [30]:

$$k_{virus}=\frac{k_{total}\times k_{cantilever}}{k_{cantilever}-k_{total}}$$

F-Z curves of particles can be converted to Force-Indentation (F-D) curves by transforming the x-coordinate ($x_{F\text{-}Z,particles}$) with the following equation, where the $k_{substrate}$ was acquired by fitting the linear part on the F-Z curve of an AFM tip indenting a glass substrate:

$$indentation=x_{F\text{-}Z,particles}-\frac{F_{F\text{-}Z,particles}}{k_{substrate}}$$

Critical force was measured from the ordinate of a discontinuous point on an F-D curve, and critical indentation was measured from the abscissa of a discontinuous point on an F-D curve.

The convolution of the tip with the sample causes the sample to appear much wider than its actual dimension laterally. However, the measured height of the sample represents a reliable parameter reflecting the sample’s dimension [30]. Therefore, the corrected height (${Height}_{corrected}$) was used as the size of noroVLP. The corrected height was calculated by the following equation [30], where the $F_{imaging}$ was the force used for AFM imaging and ${Height}_{measured}$ was the height of the particle measured from the particle’s height profile:

$${Height}_{corrected}={Height}_{measured}+\frac{F_{imaging}}{k_{virus}}$$

The one-way ANOVA method was used for significant analyses. Unless specified otherwise, stated errors are Standard Errors of the Mean (SEM).

### 2.6. Native Mass Spectrometry

NoroVLP samples were exchanged for 150 mM ammonium acetate at pH 7.4 and 250 mM ammonium acetate at pH 7.0 using Vivaspin500 centrifugal concentrators (10,000 MWCO, Sartorius, Göttingen, Germany), respectively. Measurements were performed at approximately 20 µM VP1. Native MS measurements were performed on a quadrupole time-of-flight (QToF) instrument, Q-Tof 2 (Waters, Manchester, UK, and MS Vision, Almere, the Netherlands), modified for high-mass experiments [35]. Capillaries were made by pulling borosilicate glass tubes (inner diameter 0.68 mm, outer diameter 1.2 mm with filament, World Precision Instruments, Sarasota, FL, USA) using a two-step program in a micropipette puller (Sutter Instruments, Novato, CA, USA) with a squared box filament (2.5 × 2.5 mm). Subsequently, capillaries were gold-coated using a sputter coater (Quorum Technologies, East Sussex, UK, 40 mA, 200 s, tooling factor of 2.3, end bleed vacuum of 8 × 10^−2^ mbar argon, or Safematic, Zizers, Switzerland, process pressure 5 × 10^−2^ mbar, process current 30.0 mA, coating time 100 s, 3 runs to vacuum limit of 3 × 10^−2^ mbar Argon). Ions were introduced into the vacuum at a source pressure of 10 mbar using a nanoESI source and positive ion mode. Voltages of 1.45 kV and 165 V were typically applied to the capillary and cone, respectively, and adjusted for spray optimization. Xenon was employed as a collision gas at a pressure of 1.7 × 10^−2^ mbar in order to improve transmission of high-mass ions [36]. The repetition frequency of the pusher pulse and MS profile were modified for a high mass range. A cesium iodide spectrum was recorded the same day for instrument calibration. MassLynx V4.1 SCN 566 (Waters, Manchester, UK) was used for analysis.

## 3. Results

### 3.1. Different Types of Assemblies and Sizes among Three Variants under Neutral Condition

NoroVLPs were immobilized through weak attractive interactions between particles and the hydrophobic substrate that do not change the integrity of the noroVLP capsid structure. The outer protruding arches and inner spherical shell of particles were observed both in AFM images and the corresponding height profiles (Figure 1a,b) of the noroVLPs of all three variants.

NoroVLPs of the measured variants showed a broad size distribution with multiple peaks (the histogram in Figure 1e), indicating various assembly types under neutral conditions. To assign the noroVLPs to the corresponding assemblies (*T* = 1 or *T* = 3), the size distribution of GII.17 noroVLPs was used as a base line. The size distribution of GII.17 noroVLPs was used for the height cut-off values because the morphology of GII.17 at different pH values has been studied well and the assembly state remains unaltered [11]. Furthermore, the size distribution of GII.17 noroVLPs demonstrated a clear bimodal shape in the histogram (Figure 1e). The figure reveals that GII.17 existed in the larger *T* = 3 and the smaller *T* = 1 assemblies, in line with previously published mass spectrometry (MS) results [11] under neutral conditions. For noroVLPs of GI.1, a third subpopulation could be observed around the cut-off sizes of *T* = 3 and *T* = 1. These intermediate particles are assumed to belong to VP1_80_ assemblies, i.e., particles composed of 80 VP1 monomers [37].

Regarding GII.10, most noroVLPs were assigned to *T* = 3 assemblies, but there were some smaller particles that could represent *T* = 1 assemblies. The sizes of the *T* = 1 and *T* = 3 assemblies of the different variants were within the expected range for norovirus [11,15]. Nevertheless, there was a significant difference in size between *T* = 3 assemblies of GII.10, GII.17, and GI.1 (the box plot in Figure 1e). Among the three variants, the *T* = 3 assemblies of GII.10 were the largest particles (40.4 ± 0.4 nm). The *T* = 3 assemblies of GI.1 (37.2 ± 0.3 nm) were comparable in size to the previous reports [37,38] and the size of the *T* = 3 assemblies of GII.17 (38.1 ± 0.2 nm).

**Figure 2:**
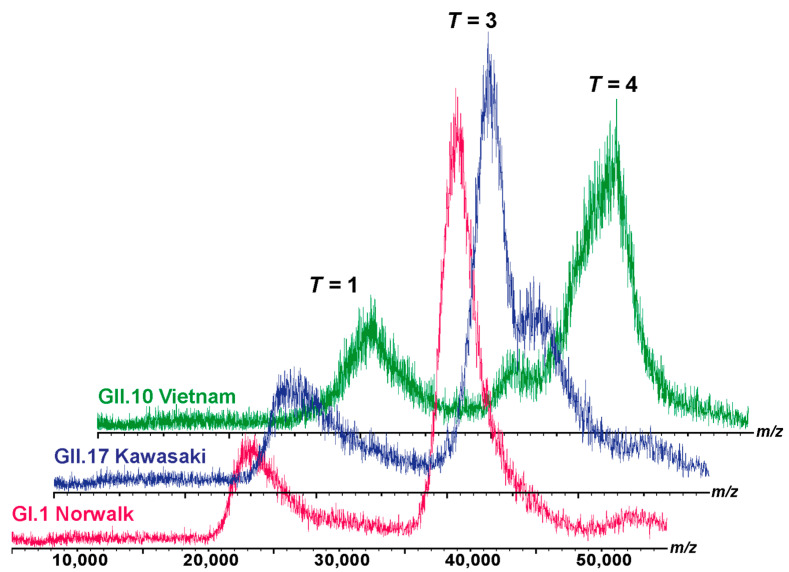
Native MS of different noroVLPs. The size distribution in 150 mM ammonium acetate at pH 7.4 at 20 µM is exemplarily shown for GI.1 Norwalk (pink), GII.17 Kawasaki (blue), and GII.10 Vietnam (green) from bottom to top. Ion distributions at approximately 23,000, 39,000, and 44,000 *m/z* were assigned to *T* = 1, *T* = 3, and putatively *T* = 4 complexes, respectively, according to the predicted charge by the Rayleigh model [39,40].

The observed assemblies are generally in line with the native MS results (Figure 2). However, discrepancies in types of assemblies may occur due to different particle concentrations and different buffer surrogate conditions [11,37] applied in the utilized methods. While AFM measurements were performed at 0.1 µM VP1, concentrations around 20 µM VP1 have to be used in native MS. The broad peak distributions, e.g., GII.10 in Figure 2, indicate the underlying dynamics of imperfect or destabilized assemblies, which may not persist at lower concentrations.

The GI.1 and GII.17 variants, when subjected to native MS analysis under 250 mM and 150 mM ammonium acetate, did not exhibit noticeable differences, as illustrated in Figure 2 and Figure S7. However, due to the restricted yield and stability of the GII.10 variant, only measurements at 150 mM ammonium acetate were performed. So different buffer surrogate conditions could also affect the types of assemblies.

### 3.2. Stepwise Deformation of noroVLPs under Compression

**Figure 3:**
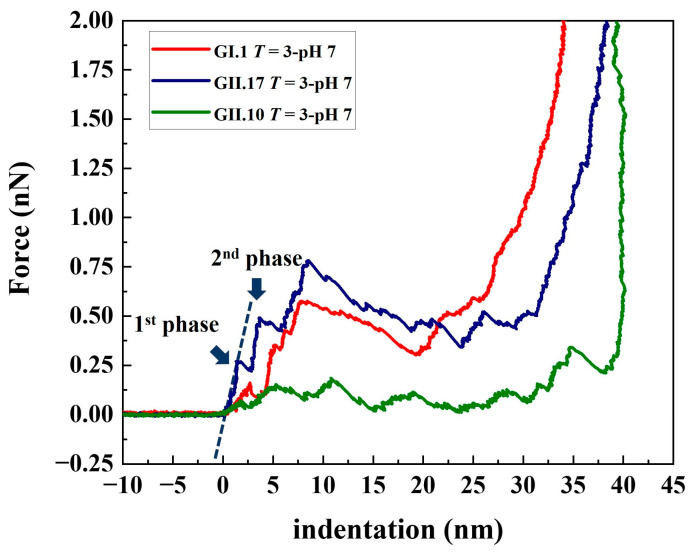
Typical approach lines of the F-D curves of the tested norovirus variants at pH 7.0. The viral spring constant of each linear phase was calculated by linear fitting at each linear phase (indicated by dashed lines in the graph). The critical force and corresponding critical indentation of each linear phase were measured from each discontinuous point (indicated by navy blue arrows in the graph) on the F-D curves. In the follow-up analysis, the first two linear fitting slopes and the ordinates and abscissas of the first two discontinuous points were included for the statistical analysis.

The deformation of noroVLPs under compression during the nanoindentation experiments followed a stepwise manner (Figure 3 and Figure S1). At the beginning of a nanoindentation cycle, the protruding arches contacted the AFM tip and were pressed together. Then the rest of the particle gradually deformed with increasing loading force. The approximate linear indentation segments of the F-D curves were separated by significant drops in force, as shown in Figure 3. These discontinuous points are associated with structural failures under the applied force, whereby the critical forces and indentations can be acquired by measuring their ordinates and abscissas. The linear parts between two discontinuous points reflect elastic deformation, and the corresponding spring constant for each linear part can be calculated following Hooke’s law. In Figure 3, the resulting linear fit is marked with a dashed line.

The F-D curves typically show a sharp increase in force at the right side of the plots, indicating that the AFM tip has reached an incompressible surface where the material beneath the tip cannot be squeezed any further. The noroVLPs of GII.10 have an average size of about 40 nm under neutral conditions. The corresponding F-D curve shows a vertical rise around 39 nm, indicating that the opposite surfaces (top and bottom) of the particle have been completely broken apart under the applied force. In contrast, the F-D curves of GII.17 and GI.1 rose vertically at around 35 nm and 33 nm, respectively. However, the average size of GII.17 is about 38 nm, and that of GI.1 is about 37 nm. Considering that the wall thickness of noroVLPs is around 2.7 nm [38], the difference between the abscissa of the F-D curve where force rose vertically and the average particle size indicates that at least one particle’s surface could withstand the applied force.

### 3.3. Different Mechanical Properties under Neutral Condition

**Figure 4:**
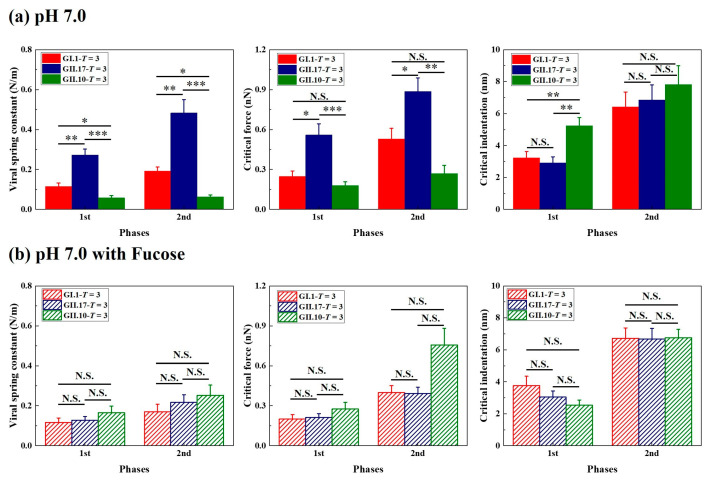
Fucose treatment diminished the original differences in the mechanical properties of the tested variants. The mechanical properties of noroVLPs at (**a**) pH 7.0 and (**b**) pH 7.0 after incubating with fucose were acquired from three independent measurements. The vertical column represents the mean, and the error bar represents the standard error of the mean. A one-way ANOVA test was used for significance analysis. *p* values are indicated by asterisks: *p* < 0.001 (***), *p* < 0.01 (**), *p* < 0.05 (*), and not significant (N.S.).

The viral spring constant, critical force, and critical indentation of the first two phases were included in the statistical analysis. The comparisons were carried out between particles of different variants but with the same assembly type. From the histogram of viral spring constant and critical force (Figure 4a), differences could be found between the measured variants, especially between GII.17 and GII.10, which was unexpected because both variants belong to the same genogroup. Among the measured variants, the noroVLPs of GII.17 are the stiffest and most robust particles, and the noroVLPs of GII.10 are the softest and most fragile particles. The mechanical properties of the *T* = 1 assemblies of GI.1 and GII.17 also show a similar relationship in that the *T* = 1 assemblies of GII.17 have a higher spring constant and critical force (Figure S2).

In contrast to the critical force and spring constant, we can find a significant difference in critical indentation exclusively at the first phase of the F-D curves (Figure 4a). No significant differences existed in the second phase. In the first phase, the critical indentations of GI.1 and GII.17 were significantly less than the critical indentations of GII.10. The difference in the first critical indentation between GII.10 (*T* = 3 assemblies of GII.10: 5.2 ± 0.5 nm) and the other two variants (*T* = 3 assemblies of GI.1: 3.2 ± 0.4 nm; *T* = 3 assemblies of GII.17: 2.9 ± 0.4 nm) was around 2 nm.

### 3.4. Fucose Treatment Diminished Original Differences

Regarding the measured variants, fucose treatment did not change the assembly types of each variant but had an effect on the particle size of GII.17 and GII.10 (Figure S6b,c). On the contrary, fucose treatment did not affect particle sizes of GI.1 (Figure S6a). Fucose treatment changed the viral mechanical properties of measured variants in different ways. For noroVLPs of GII.17, fucose treatment significantly decreased their viral spring constant and critical force but did not significantly affect their critical indentation (Figure S4a). In contrast, fucose treatment significantly increased the viral spring constant and critical force of GII.10 noroVLPs and decreased their critical indentation (Figure S5). For GI.1, fucose treatment showed no significant effects on viral spring constant, critical force, or critical indentation (Figure S3a,b). As a result of the different responses of the measured variants to fucose treatment, the previously observed significant differences in mechanical properties between the three variants were diminished (Figure 4b).

### 3.5. Alkaline Treatment Weakened Mechanics of noroVLPs

**Figure 5:**
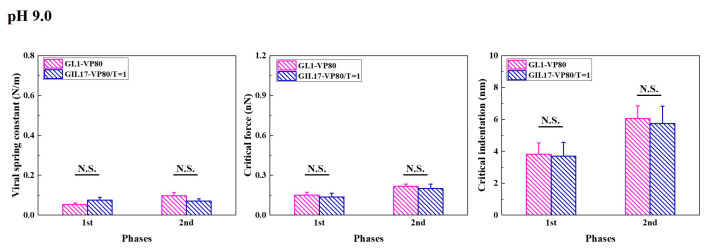
Alkaline treatment affected the mechanical properties of GI.1 and GII.17, leading to a similar mechanical performance of the two variants. The mechanical properties of noroVLPs of GI.1 and GII.17 at pH 9.0 were acquired from three independent measurements. The vertical column represents the average, and the error bar represents the standard error of the mean. A one-way ANOVA test was used for significance analysis. No significant (N.S.) indicates the *p* value is larger than 0.05.

Increasing the pH value to 9.0 decreased *T* = 3 assemblies of GI.1 and enlarged the sizes of *T* = 1 and *T* = 3 assemblies of GII.17 (Figure S6). At pH 9.0, some small particles of GII.17 fell within the size range of the VP1_80_ assembly, previously defined based on the noroVLP size distribution under neutral conditions. However, as *T* = 3 also increased in size, we cannot confirm whether some of the small particles of GII.17 that we found at pH 9.0 belong to VP1_80_ or *T* = 1 assemblies. Hence, the histograms of GII.17 in Figure 5 are noted as VP1_80_/*T* = 1. Except for viral spring constants, alkaline treatment had no significant effects on other mechanical properties of VP1_80_ assemblies of GI.1 (Figure S3b). For GII.17 and *T* = 1 assemblies of GI.1, alkaline treatment significantly reduced their viral spring constant and critical force but did not significantly affect their critical indentation (Figures S3c and S4). In general, alkaline treatment significantly lowered the viral spring constants of GI.1 and GII.17, leading to a similar mechanical performance of the two variants in the end (Figure 5).

## 4. Discussion

In this study, we measured noroVLPs of the prototypical strain GI.1 Norwalk, the Asian epidemic strain GII.17 Kawasaki 308, and the strain with a broad binding profile, GII.10 Vietnam 026, under different experimental conditions. The size variations between the three variants are likely caused by different conformations of VP1 monomers. Compared with the P domain of prototypical GI.1, the P domain of GII.10 significantly lifts (∼15 Å) from the S domain through a flexible hinge region [15]. Correspondingly, the difference in size found in our AFM measurements between noroVLPs of GI.1 and GII.10 under neutral conditions was about 3.2 nm, which matches the expected size enlargements due to a risen P domain. However, it is unclear if the rising conformation of the P domain is a universally shared feature among genogroup II strains [41]. For instance, despite belonging to the same genogroup, there was a difference in size between the *T* = 3 assemblies of GII.17 and GII.10.

**Figure 6:**
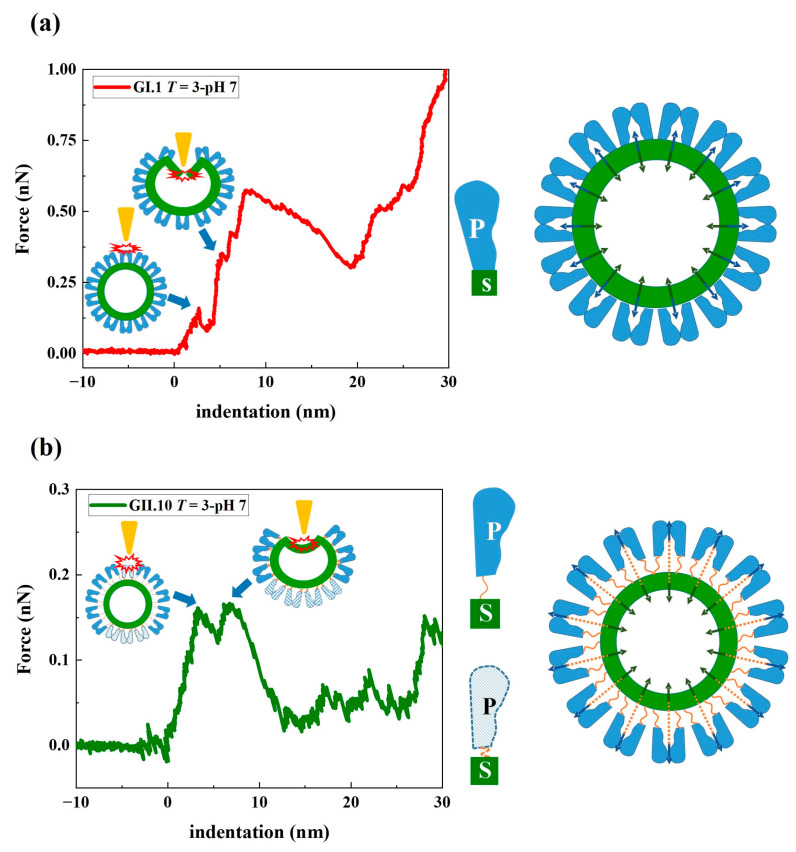
Mechanical model of (**a**) GI.1 and (**b**) GII.10 noroVLP deformation under the applied force. The rising conformation of the P domain of GII.10 capsid protein VP1 is depicted by the unfolded hinge connecting the P and S domains (indicated by the orange string in (**b**)). The navy blue arrows in the model indicate the tension pointing outward generated by the dimeric interactions between P domains. The dark green arrows indicate the inward force of the S domain acting on the P domain, thereby creating a balanced state of forces in the capsid. In the representative F-D curves of noroVLPs of GI.1 and GII.10, the corresponding events on particles when the first two discontinuous points happened are depicted and indicated by blue arrows.

While applying force by AFM, the stepwise deformation of noroVLPs agrees well with coarse-grained structure-based simulations of nanoindentation on norovirus capsids [42]. Compared to the required force for deforming and destructing the protruding arches directly above the shell, less force and more tip engagement are required to destruct a floating protruding outer layer, resulting in lower 1st viral spring constants and higher 1st critical indentations. The difference in the 1st critical indentation between GII.10 and the other two variants (~2 nm) was roughly equal to the distance between the P domain and the S domain found in the cryo-EM map of GII.10 VLP (EMD-5374). It supports our hypothesis that the first phase on F-D curves reflects the local mechanical states of protruding arches and represents the required tip engagement to deform the protruding arches. Referring to the prestress model, as shown in Figure 6, the norovirus capsid is stabilized by the outward tension generated from P-dimeric interactions and the inward force for keeping the shell at the ground state [38]. The flexible hinge region between the P and S domains could modulate the ratio of tensions pointing outward and inward. So the extended hinge region acts as a cushion, absorbing some of the outward tension applied to the inner shell. Therefore, noroVLPs of GII.10, having an extended hinge region, show the lowest 2nd viral spring constant.

Current viral identification methods are continuously challenged by the capability of viruses to mutate and the growing demands for early therapy and epidemic prevention [19]. Methods for recognizing specific targets, like polymerase chain reaction (PCR) and immunoassays, can miss variants due to virus evolution under immune pressure [43]. Although electron microscopy (EM) is not target-directed, it is optimized for static conditions. Therefore, there is still a need to develop new identification methods that can overcome the constraints of these current methods. AFM-based imaging and nanoindentation techniques show high specificity in differentiating norovirus genotypes that are even from the same genogroup. Thus, characterization of viral mechanical properties could be used in combination with other methods to provide a more rapid and highly specific identification of viruses and their mutates.

During host infection, norovirus passes through the stomach with an acidic pH and is finally confronted with a higher pH at the small intestine, where norovirus enters the host cells [16]. Increasing pH lowered the mechanical strength (critical force) and significantly reduced viral stiffness (viral spring constant) for GI.1 and GII.17 variants, which are logical changes preparing viruses for endocytosis and uncoating. While the significant differences observed under neutral conditions were smoothed out in alkaline conditions, slight differences in stability can still be found in their assembly types. *T* = 3 assemblies of GI.1 are lacking at pH 9.0. In contrast, although size increased, both *T* = 3 and *T* = 1 assemblies of GII.17 remained intact. These AFM results agree with the assemblies observed by native MS under similar conditions [11,37]. Moreover, the alkaline pH-induced size increase was in line with a previous report [44].

In the small intestine, the fucose moieties of HBGAs serve as the primary determinant for norovirus recognition. The initial differences in mechanical properties between the variants were diminished upon fucose treatment. Fucose treatment showed no significant effects on the mechanical properties of GI.1. On the contrary, fucose treatment demonstrated converse effects on the two variants from genogroup II. GII noroviruses bind HBGAs at their dimeric interface, while GI.1 binding sites are located on a single dimeric subunit [45]. It suggests that fucose binding on a single dimeric subunit, as for GI.1, is insufficient to affect the mechanical properties of noroVLPs.

Within genogroup II, the opposite responses to fucose binding reveal strain-dependent structural dynamics. In hydrogen-deuterium exchange (HDX) MS, only the canonical binding site is usually protected from exchange. When a GII.17 P dimer binds to fucose, however, residues 269–286 also become protected. In GII.10, on the other hand, residues 311–336 become protected in addition to the binding site, which is attributed to the fucose-induced folding of a small loop into an α-helix proximal to the binding site [46]. As these are not direct glycan binding sites, the protection at these sites could be due to long-distance structural alterations rather than direct fucose binding. Control experiments using 100 mM D-galactose, which is not expected to bind norovirus, showed no effect in HDX-MS [33,46]. Therefore, the changes in the measured variants are also unlikely to result from unspecific effects of fucose. As a result, it is possible that the fucose-induced structural changes propagate into the flexible hinge region, resulting in larger GII.17 particles with less stiffness by lifting the P domain above the S domain. In contrast, fucose induces opposite changes at the flexible hinge region of GII.10, causing size reduction and increased viral spring constant and critical force.

As above described, fucose binding clearly has a strain-dependent effect on the mechanical properties, suggesting it is not only an attachment factor but also has an impact on structural dynamics via a flexible P domain. The flexibility of the P domain is conserved across the family of noroviruses [17], indicating that P domain flexibility is crucial during the viral life cycle, for instance, during cell entry. However, the exact function of P-domain flexibility is still under debate with conflicting reports in the literature. In a recent study, Song et al. [16] found that murine norovirus having the P domain at rest demonstrated higher affinity and infection rate to the host cells compared to the murine norovirus with rising P domain conformation. This could indicate that GII.10 found a way in which binding of fucose increases infection by triggering the transformation to the resting conformation. This raises the question of why fucose binding to GII.17 has the opposite effect. Hu and colleagues [18] proposed that the resting conformation protects antigenic sites on the capsid surface and that the rising conformation allows for the binding of a cofactor. Though the latter still requires more evidence, it is conceivable in an evolutionary context. The prototypical GI.1 has so far only shown a resting conformation, suggesting that the P domain capsid dynamics are a more recent development. Floating P dimers render the virus more vulnerable to an immune response. Therefore, it is reasonable to assume that this comes with an advantage, such as serving as a ruse to confuse the immune response by exposing non-neutralizing epitopes or releasing soluble P domains that have been cleaved by proteases [17].

In summary, we used AFM nanoindentation to measure the noroVLPs of three distinct human norovirus variants. The observed differences in viral mechanical properties across the studied variants under neutral conditions are reflected by the various conformations of the norovirus capsid protein. While the measured variants may not be fully representative of their corresponding genotypes, the notable differences in viral mechanical properties among these variants provide impetus to investigate multiple strains within each genotype, thus elucidating the genotype-specificity in the following research. The measured viral physical properties have the potential to serve as novel indications for differentiating virus variants. We revealed that the norovirus variants exhibited varied reactions to fucose binding, and we highlighted the effects of the distinct responses on the viral physical properties using AFM measurements. The fucose treatment diminished the original variations in viral mechanical properties between the norovirus variants. The observed fucose-induced differences hint at different structural changes in the norovirus capsid protein VP1. This suggests that various strategies are applied by noroviruses to improve their infectivity and immune evasion.

## Data Availability

The data presented in this study are available on request from the corresponding author.

## Supplementary Materials

The following supporting information can be downloaded at: https://www.mdpi.com/article/10.3390/v15071482/s1, Figure S1: Typical approach lines of F-D curves of noroVLPs; Figure S2: Different mechanical properties of *T* = 1 noroVLPs of GI.1 and GII.17 at pH7.0; Figure S3: The effects of different experimental treatments on the mechanical properties of *T* = 3, VP1_80_, and *T* = 1 of GI.1; Figure S4: The effects of different experimental treatments on the mechanical properties of *T* = 3 and VP1_80_/*T* = 1 of GII.17; Figure S5: The effects of different experimental treatments on the mechanical properties of *T* = 3 assemblies of GII.10; Figure S6: The comparison between corrected heights of GI.1, GII.17 and GII.10 under different experimental conditions; Figure S7: Native MS of noroVLPs in 250 mM ammonium acetate.
