# Supplementary material for: Fucose Binding Cancels out Mechanical Differences between Distinct Human Noroviruses
Source: Viruses. 2023 Jun 30;15(7):1482. doi: 10.3390/v15071482 (PMC10383637; doi:10.3390/v15071482)
Supplement: Supplementary file 1 [file viruses-15-01482-s001.zip › viruses-2377970-supplementary.pdf]

Supplementary Material

Fucose binding cancels out mechanical differences between distinct human noroviruses

Yuzhen Feng [1], Ronja Pogan [2,3], Lars Thiede [2,3], Jürgen Müller-Guhl [2,4], Charlotte Uetrecht [2] and Wouter H. Roos [1,]*

1 Moleculaire Biofysica, Zernike Instituut, Rijksuniversiteit Groningen, the Netherlands
2 CSSB Centre for Structural Systems Biology, Deutsches Elektronen-Synchrotron (DESY) & Leibniz Institute of Virology (LIV), 22607 Hamburg, Germany
3 University of Siegen, Faculty V: School of Life Sciences, 57076 Siegen, Germany
4 Partner Site Hamburg-Lübeck-Borstel-Riems, Bernhard Nocht Institute for Tropical Medicine and German Center for Infection Research (DZIF), 20359 Hamburg, Germany
* Correspondence: w.h.roos@rug.nl

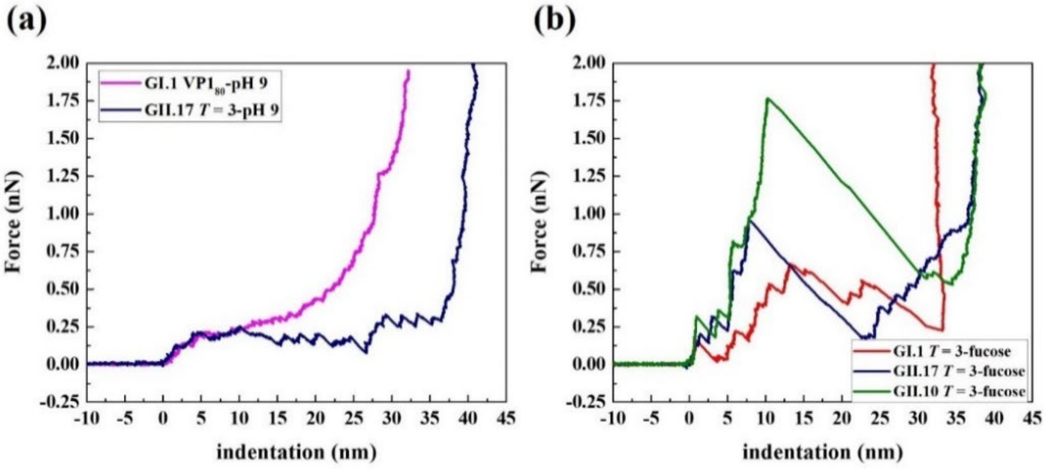

Figure S1. Approach lines of the F-D curves of the noroVLPs upon (a) alkaline and (b) fucose treatments. The deformations observed in alkaline-treated and fucose-treated noroVLPs during nanoindentation displayed a stepwise feature, similar to the deformations when noroVLPs were compressed under neutral conditions.

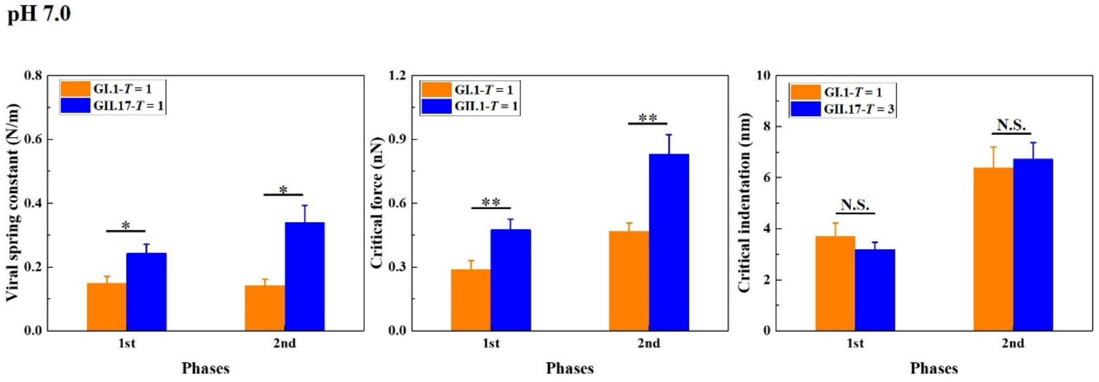

Figure S2. Differences in mechanical properties between the T = 1 assemblies of the tested variants. The mechanical properties of the T = 1 assemblies of GI.1 and GII.17 at pH7.0 were

acquired from three independent measurements. The vertical column represents the average and the error bar represents the standard error of the mean. One-way ANOVA test was used for significance analysis. P values are indicated by asterisks: p<0.01 (**), p<0.05 (*) and not significant (N.S.).

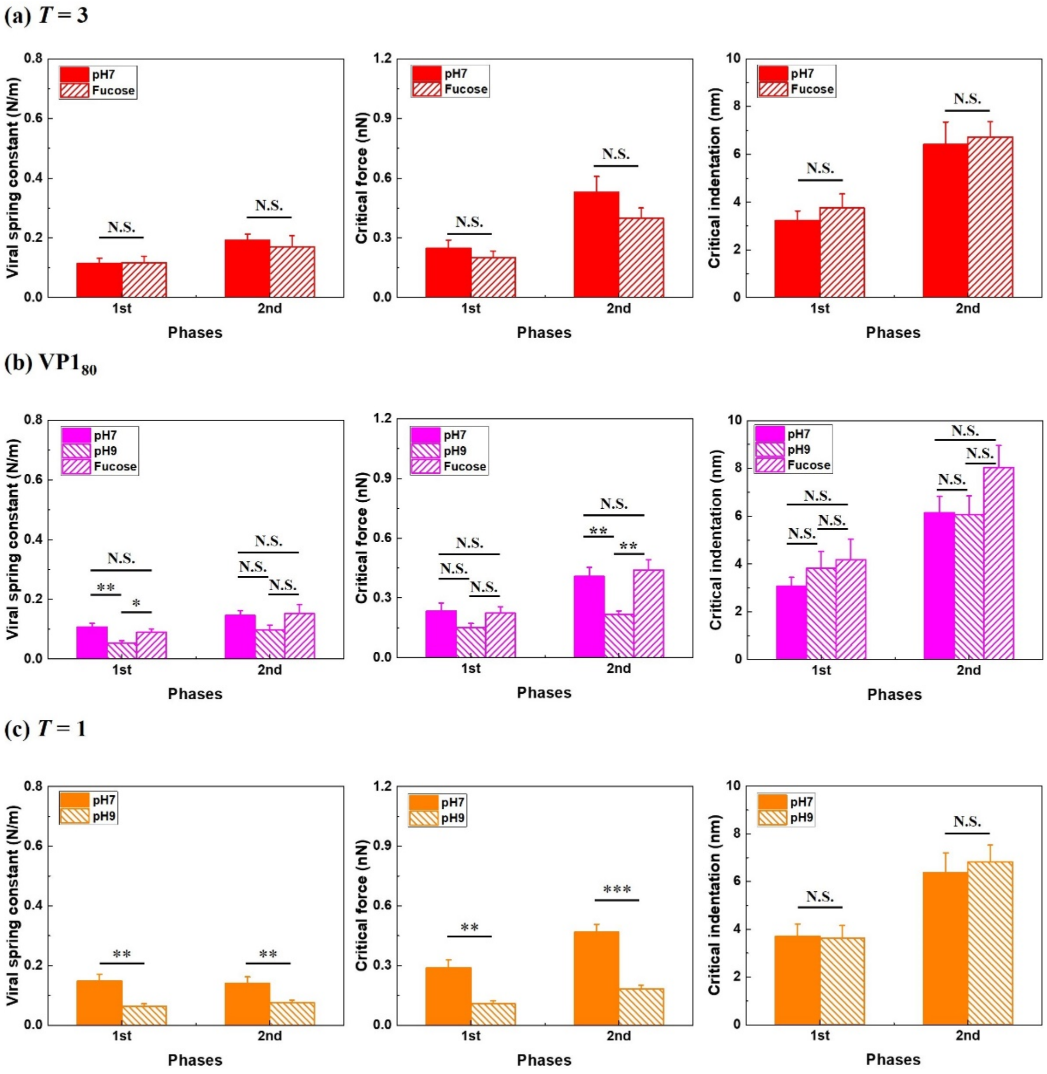

Figure S3. The effects of alkaline and fucose treatments on the mechanical properties of the (a) *T* = 3, (b) VP1$_{80}$, and (c) *T* = 1 assemblies of GI.1. The data sets were acquired from three times independent measurements. The vertical column represents the average and the error bar represents the standard error of the mean. One-way ANOVA test was used for significance analysis. P values are indicated by asterisks: p<0.001 (***), p<0.01 (**) and not significant (N.S.).

(a) $T = 3$

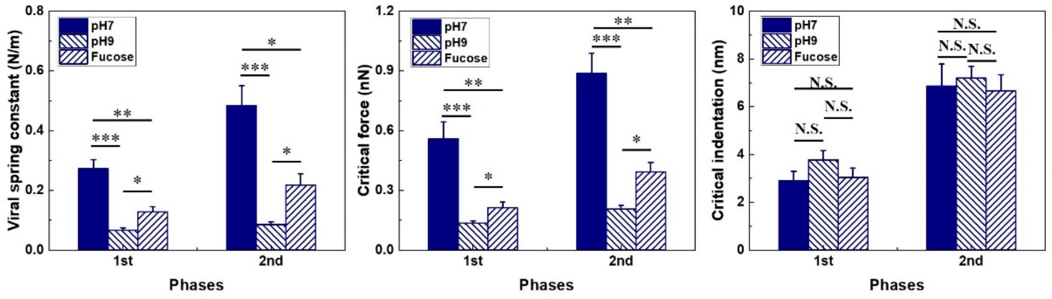

(b) $VP1_{80}/T = 1$

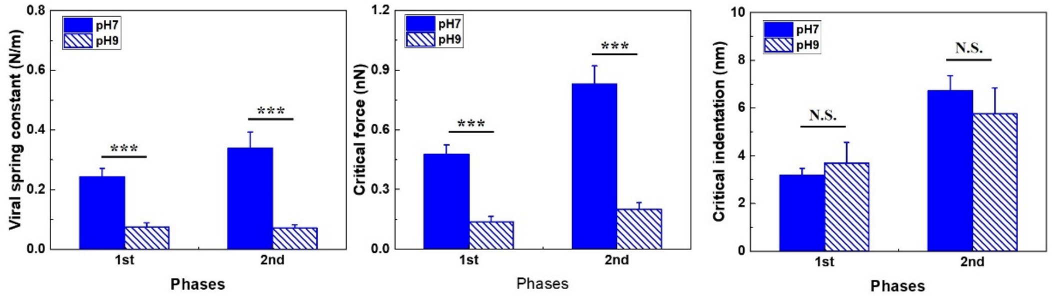

Figure S4. The effects of alkaline and fucose treatments on the mechanical properties of the (a) $T = 3$ and (b) $VP1_{80}/T = 1$ assemblies of GII.17. The data sets were acquired from three times independent measurements. The vertical column represents the average and the error bar represents the standard error of the mean. P values are indicated by asterisks: p<0.001 (***), p<0.01 (**), p<0.05 (*) and not significant (N.S.).

$T = 3$

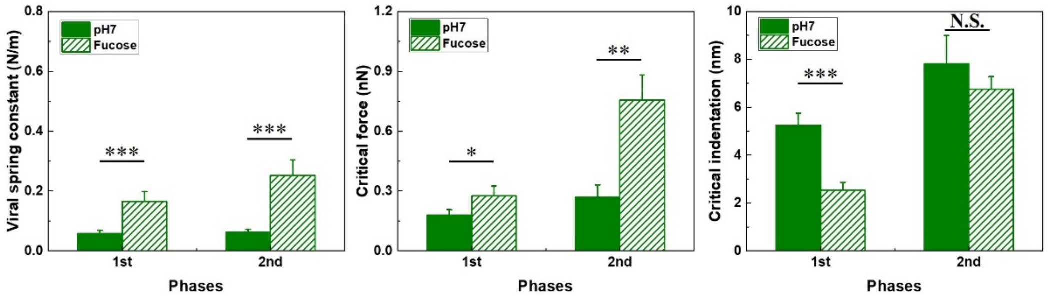

Figure S5. Fucose treatment changes the mechanical properties of the $T = 3$ assemblies of GII.10. The data sets were acquired from three times independent measurements. The vertical column represents the average and the error bar represents the standard error of the mean. One-way ANOVA test was used for significance analysis. P values are indicated by asterisks: p<0.001 (***), p<0.01 (**), p<0.05 (*) and not significant (N.S.).

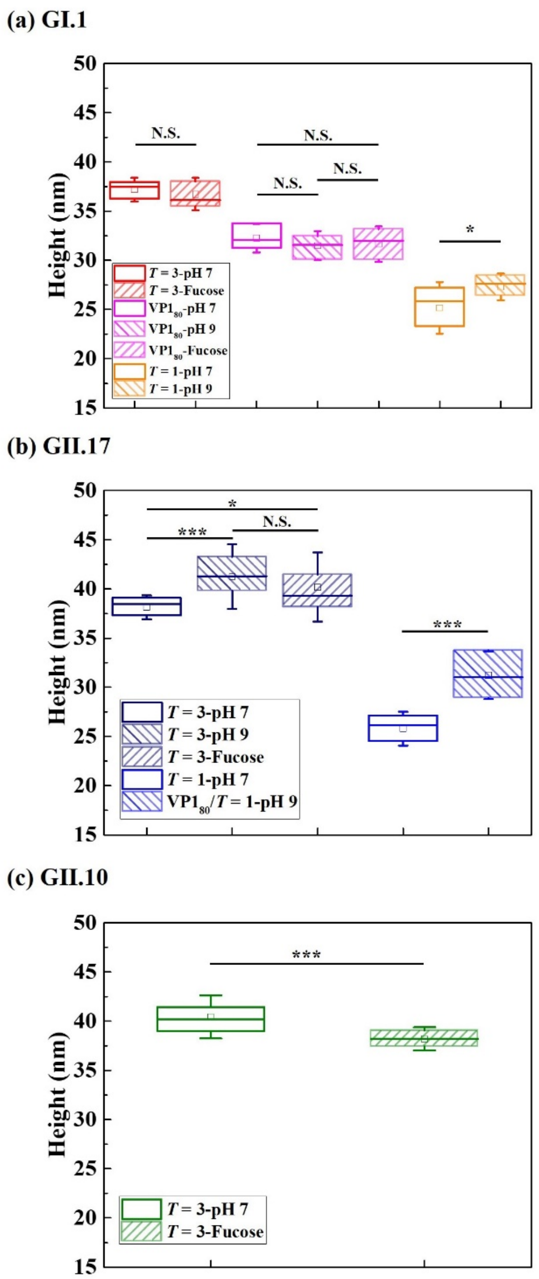

Figure S6. The effect of alkaline and fucose treatments on the size of the noroVLPs of (a) GI.1, (b) GII.17 and (c) GII.10. The data sets were acquired from three times independent measurements. The whiskers in the box plot represent the standard deviation. One-way ANOVA test was used for significance analysis. P values are indicated by asterisks: $p<0.001$ (***), $p<0.01$ (**), $p<0.05$ (*) and not significant (N.S.).

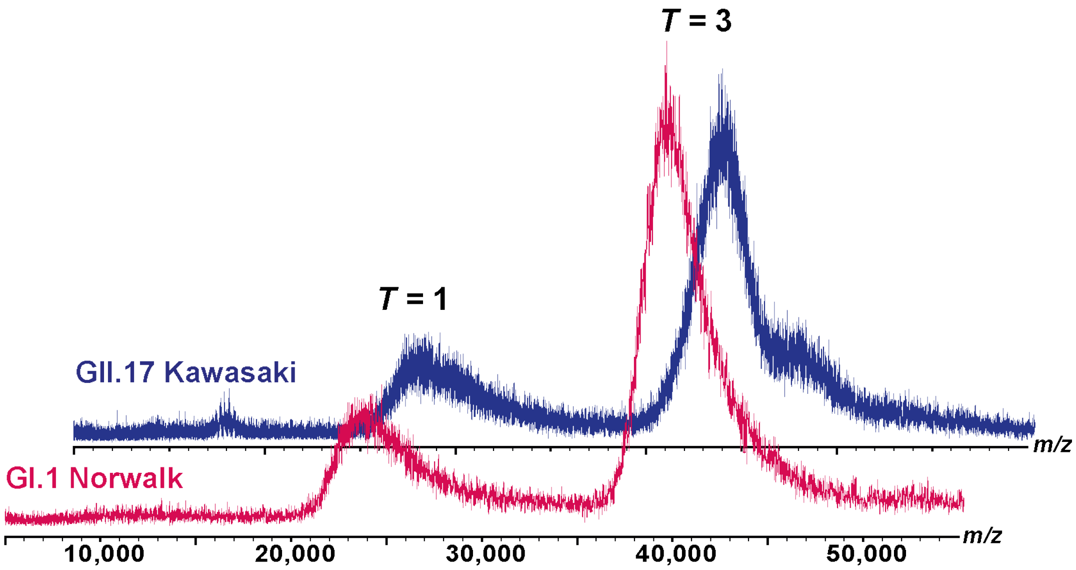

Figure S7. Native MS of noroVLPs in 250 mM ammonium acetate. The size distribution in 250 mM ammonium acetate at pH 7.0 at 25 µM is exemplarily shown for GI.1 Norwalk (pink) and GII.17 Kawasaki (blue). Due to low yield and limited stability, GII.10 Vietnam is not available in 250 mM ammonium acetate. Ion distributions at approximately 23,000 and 39,000 *m/z* were assigned to $T = 1$ and $T = 3$ complexes, respectively.
